# Levels of Some Heavy Metals in Raw Cow's Milk from Selected Milk Production Sites in Iran: Is There any Health Concern?

**DOI:** 10.15171/hpp.2015.021

**Published:** 2015-10-25

**Authors:** Mohamadreza Arianejad, Mohammad Alizadeh, Arash Bahrami, Seyed Rafie Arefhoseini

**Affiliations:** ^1^Department of Food Science and Technology, Faculty of Nutrition, Tabriz University of Medical Science, Tabriz, Iran; ^2^Tabriz Health Services Management Research Center, Tabriz University of Medical Sciences, Tabriz, Iran; ^3^Department of Nutrition, Faculty of Nutrition, Tabriz University of Medical Science, Tabriz, Iran

**Keywords:** Heavy Metals, Milk, Atomic Absorption, Iran

## Abstract

**Background:** The aim of this study was to evaluate the content of mercury (Hg), arsenic (As), nickel (Ni) and tin (Sn) in raw cow’s milk of traditional and industrial sites from 8 different sites in Arak City, Markazi Province, Iran.

**Methods: ** In this cross sectional study, a total of 32 samples were collected from sub-cities of Arak, Iran via subjective sampling method. Both industrial and traditional dairy farms were selected for sampling. Twenty-five gram of each sample was turned to ash in ovens for metal analyses including Hg, As, Ni and Sn by anatomic absorption spectrometer.

**Results:** The residue amounts of Hg and As were lower than permissible limit suggested by Codex Alimentarius, but for Ni and Sn it was higher only in one of the collection sites. The average concentra­tion of Hg was significantly higher (P<0.05) in traditional farms as compared to industrial farms. Be­sides, amounts of Sn was significantly high in the traditional farms (P<0.05). Further, a high contents Ni was detected in industrial farms (P<0.05).

**Conclusion:** High Sn and Ni contents of some milk samples from this region might be potentially hazardous to consumers. Further, none of the other metals tested crossed permissible levels.

## Introduction

Dairy products and in particular milk contain variety of very important nutrients which are crucial to maintain healthy life of every individual. Thus, their regular daily consumption has been widely recommended. However, there is evidence that milk and other dairy products might contain varying amounts of different toxic contaminants. According to the newest report from WHO one in eight global deaths were linked with air pollution, making it "the world's largest single environmental health risk".^1^ Thus, it is a vital topic to study pollutant particles such as heavy metals in the air polluted areas. Human exposure to these compounds occurs in different ways including inhalation, dermal contact and via food items with the later accounting for at least 90% of overall human exposure.^2^ Therefore, the contamination of living environment with potentially toxic heavy metals is considered as a very important health concern, which may result in accumulation of the elements in many food items.

The cow’s milk is utilized all over Iran to produce many dairy products, which implies ensuring safety of this important food items. Heavy metals have been named in this attribute because of their high atomic weight. Mercury (Hg), arsenic (As), nickel (Ni) and tin (Sn) belong to class of heavy metals, which are capable to permeate in foods and thereby enter the body. If contamination continues, it results in aggregation into body and may cause acute or chronic intoxication particularly among children and adolescents.^3^ Thus, the amount and composition of heavy metals in different foods is always the focus of study for many researchers.^4-6^

Degree of dietary exposure to either toxic or nutrient elements is attributed to the amount of food together with processing technology and especially condition from the farm to the production.

Arak City, central Iran is a polluted city of central Iran with a large number of industrial units, as in many days of year the concentration of pollutants in its atmosphere cross the warning limit. This high level of pollution is expected to contaminate soil, water and more specifically agricultural lands. For this reason, in this area feeding the cows accompanies with potential risk of heavy metals accumulation in bodies of animals and collectively in their milks.

The aim of this research was to determine and compare amount of Hg, As, Ni and Sn in raw cow’s milks from traditional and industrial sites.

## Materials and Methods

### Collection of samples

In this cross sectional study, four sub cities of Arak City, central Iran were selected using subjective sampling method. Thirty-two samples (eight samples from each selected area) were collected from both industrial (n=16) and traditional (n=16) dairy farms from August 2014 to November 2014. The sampling polyethylene container was kept overnight in nitric acid and rinsed with double distilled water to avoid any contamination. A total of 250 ml of milk samples were collected from freshly filled milk containers of each farm and placed in an icebox. The samples were frozen within an hour at -20 °C until analysis.

### Sample preparation

Twenty-five gram of each sample was weighed in ceramic crucibles and dried in 450 °C by heater. Then the crucibles were put on a flame and burnt. After that, crucibles containing the samples were put in the oven at 450 °C for 4 hours until the sample turned to ash. In the next step, 0.1 mol of nitric acid was added to the vessel containing the sample. Then it was flattened in a balloon and the volume was increased to 50 ml with nitric acid 0.1 molar. In the next step, 20 ml of each sample was transferred into a funnel decanter (separation), few drops of Bromocresol were added to the detector, and eventually 4 ml of citric acid was added to it. The samples pH was regulated on 5.4 by ammonia.

### Analysis of metals

The analysis of Hg, As, Ni and Sn was performed by a Varian AA230 atomic absorption spectrometer with a graphite Furnace (GTA 340). The spectrometric condition for the analysis is summarized in Table 1.

### Statistical analysis

Concentrations were expressed as mean ± SD. Experimental results were evaluated by paired *t* test, ANOVA and frequency distribution method with SPSS, version 16.0 for windows (SPSS Inc., Chicago, USA).

### Ethical Considerations

The research proposal was reviewed and approved by Deputy for Research Affairs at Tabriz University of Medical Science, Tabriz, Iran. All the study procedure was conducted in accordance to national research ethic guidelines.

**Table 1:** Conditions for Graphite Furnace – Atomic Absorption Spectrometry

**Metal**	**Wavelength (nm)**	**Pre- Heating (° C)**	**Drying (° C)**	**Ashing(° C)**	**Atomization (° C)**
Mercury	253.7	75	125	1550	1800
Arsenic	193.7	80	120	1600	2000
Nickel	221.5	70	120	1700	1900
Tin	198.5	65	110	1650	1850

## Results

Table 2 shows range of the heavy metals, permissible limits, provisional tolerable weekly intake (PTWI) and estimated intake values of Hg, As, Ni and Sn by frequency distribution method. The contents of Hg and As in the tested samples were in the ranges from 7.29 to 14.95 µg/l and 15.20 to 25.90 µg/l, respectively. All the values were within permissible limits set by Codex Alimentarius and PTWI.^7^ Estimated daily intakes of the two heavy metals, assuming that annual 150 liters of milk per capita consumption in the region was 4.50µg/day for Hg and 8.52 µg/day for As (Table 2). Analysis of Ni and Sn contents in the samples yielded a minimum values of 45.10 (µg/l) and 29.78 (µg/l) and maximum values of 310.55 (µg/l) and 314.64 (µg/l), respectively. In one of the sample collection sites amount of Sn slightly crossed permissible limit. Besides, daily intakes of the Ni and Sn from milk consumption were estimated at 34.92 µg and 28.23 µg, respectively.

**Table 2:** The values of Mercury, Arsenic, Nickel and Tin

**Estimated Intake** **(µg/d)** ^**^	**PTWI** ^*^ **(µg/d/60 kg)**	**Permissible limit by Codex Standard** **(µg/l)**	**Range** **(µg/l)**	**Number of samples**	**Metal**
4.50	300	500	7.29 -14.95	32	Hg
8.52	128	140	15.20 - 25.90	32	Arsenic
34.92	ND^***^	200	45.10 -310.55	32	Nickel
28.23	ND^***^	300	29.78 -314.64	32	Tin

^*^PTWI provisional tolerable weekly intake, ^**^ Assuming that Per Capita Consumption of milk in HGArak is about 150 liter ^***^ND not determined

**Fig. 1:**
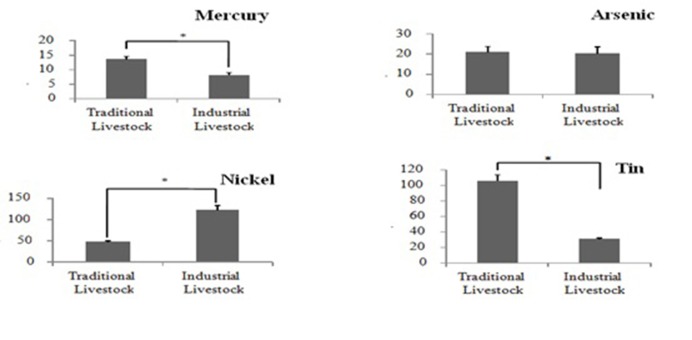
Comparison of mercury, arsenic, nickel and tin at traditional and industrial livestock. A single asterisk represents *P* value less than 0.05 using student’s *t*-test

Mean elemental comparison of milk from both traditional and industrial farms is presented in Figure 1.The average concentration of Hg was significantly higher (*P*<0.05) in traditional farms as compared to industrial farms. Moreover, the amounts of Sn was significantly higher in the traditional farms (*P*<0.01). Further, high amounts of Ni was determined in industrial farms as compared to the traditional farms (*P*<0.05). There was no difference in concentrations of As between the two farms.

Table 3 shows results of the correlation analysis between the four elements tested. There were no significant correlations between concentrations of Sn with either Hg (*P*=0.38, r=0.36) or As (*P*=0.38, r=0.36). No correlation was observed between concentrations of Ni with that of As (*P*=0.46, r=0.31). However, a strong negative correlation was determined between concentrations of Hg and Ni (*P*=0.01, r=-0.83) and a no correlation between concentration of Ni and Sn (*P*=0.94, r=-0.36). Finally, no correlation existed between concentrations of As with that of Hg (*P*=0.48, r=0.12).

**Table 3:** Correlation* between mercury, arsenic, nickel and tin

	**Mercury**		**Arsenic**		**Nickel**	
Item	**r**	***P***	**r**	***P***	**r**	***P***
Arsenic	0.12	0.48	-	-	-	-
Nickel	- 0.83	0.01	0.31	0.46	-	-
Tin	0.36	0.38	0.43	0.29	- 0.36	0.94

* Classified by Spearman rank

Comparisons for the average levels of the four heavy metals between traditional and industrial livestock are presented in Figure 1. The average concentrations of Hg and Sn at the traditional livestock was significantly higher (*P*<0.05) than industrial livestock. In contrast, at the industrial livestock Ni value was significantly higher (*P*<0.05) than the traditional livestock. Whilst the amount of as in two types of livestock was nearly equal.

## Discussion

Today, environmental risk factors possess a substantial contribution in the burden of disease both globally and nationally.^8^ Due to high stability in the nature and bioaccumulation of these pollutants various public health concerns are identified. Plants are able to absorb heavy metals from the soil. The plants with rich heavy metals contents can be used as animal feed. This, in turn, results in contamination of animal products such as milk. Consequently, metal residues in milk may possess further adverse effects on consumer's health and in particular failure of mental development among children and infants. In industrial areas (as in our study area), milk pollution is important due to possibility of heavy metal contamination linked to industrial units. It should be noted that milk is not the only source of absorption of these toxic metals. Therefore, amount of our research might be considered as an important challenge.

A narrative comparison of the elemental concentrations of whole milk in the current study with other studies is provided in Table 4. In comparison with the average Hg concentrations of raw milk obtained by Bilandzic et al,^5^Anastasioet al,^9^ and Caggianoet al*,*^10^ the samples in the current study had relatively higher amounts of the element. In the case of As, our findings were comparable with a report by Licataet al,^11^ but remarkably higher than the amounts detected by Ahmet et al.^6^ Further, Ni concentration was remarkably higher as compared to some of other reports,^11-17^ and lower than a report by Javadet al.^13^ Finally, Sn contents of the milk samples were extremely higher as compared to the two other reports.^18,19^

Sn is widely used in various industries. Contamination of milk by Sn depends on the geographic area, type of nearby industrial set ups and water or animal feed pollution by this metal. The higher levels of Sn in milk compared to the other studied metals may be due to the ability to bond with carbon in milk structure and subsequent formation of organic Sn which is stable and toxic.^20^ High concentrations of Ni in traditional farms compared to industrial farms could be attributed to the location of this industry in a rural area, which included the dairy livestock, and possibility of having those metals entering in the food chain.

**Table 4:** Comparison of ranges or means concentrations of Nickel, Arsenic, Nickel, Tin in milk samples with other Studies

**Tin**	**Nickel**	**Arsenic**	**Mercury**	**Reference**
29.78 -314.64	45.10 – 310.55	15.20 – 25.90	7.29 -14.95	This study
-	-	1 – 283 ( µg/lit)	1.59-7.1 (µg/lit)	(5)
-	-	-	0.002 ± 0.005 ( µg/g)	(9)
-	-	-	0.0025 ( µg/g)	(10)
-	-	0.08-0.00 (mg/kg )	-	(6)
-	-	37.90 (µg /kg )	-	(11)
-	22.395 ± 0.988 (mg/L)	-	-	(13)
-	MDL* (mg/kg)	-	-	(15)
-	0.058-1.750 (µg /g )	-	-	(16)
0.59±0.07 (µg/lit)	12±4 (µg/lit)	13.7±0.8 (µg/lit)	-	(19)
-	3.013- 2.097 (mg/L** )**	-	-	(14)
0.10 (µg/dl)	0.04 (µg/dl)	-	-	(18)
^*^Below method detection limit				

Levels of Hg and Sn in traditional sites were higher than those in the industrial livestock were; however, Ni content was higher in the industrial areas. The levels of As and Hg measured in the current study were comparable with those in others. For example in some studies, these two elements were not detected in milk samples,^21^ or its value was negligible.^22^ The findings disagrees with a report by Bilandžić et al. that levels of Hg in milk samples exceeded the maximum recommended level in both northern and southern parts of Croatia.^5^ Average levels of the element in southern regions of the country was significantly higher than northern areas.^5^ Hend et al.  reported a significantly higher amounts of Hg from row milk to cheese.^4^ There are limited data on Sn residues in raw cow milk in comparison with other heavy metals.

A lack of different concentrations of As between the two types of farms could be attributed to transportation of these compounds from one place to another by air or as a gas or as absorbed or adsorbed species on particulate material in suspension.

The lack of correlation between the heavy metals in this study would be due to different pollution sources. In the other words, the presence of one element did not significantly affect the levels of other heavy metals. The interaction of Ni and Hg can be linked to the source of these two metals in the studied areas. Generally, metal concentrations were evaluated in this study were more or less comparable with other report measurements.

This study had some limitations. It would have been better if we had examined seasonal variations in amounts of the metals since pattern of animal feeding is evidently different within the seasons. Furthermore, examining amount of the selected metals in animal feeds and even in soil and water resources of the study region could help better explanation of the results which warrants further studies.

## Conclusion

Even though milk samples tested did not contain higher amounts of minerals in reference to permitted levels, except for Sn in a selected area, the amounts of heavy metals consumed is still a matter of health concern since many other locally produced food items may have remarkable amount of contamination. In addition, in some industrial areas due to the air pollution and subsequent contamination of animal feed and waters, the levels of heavy metals in milk and other dairy products may be increasing. More specifically, high amounts of Sn might be potentially hazardous to consumers. It is suggested that control and monitoring of water and feed for livestock and application of appropriate containers in transit of raw milks may be helpful for production of healthier milks.

## Acknowledgment

This study was supported by a grant from the Tabriz University of Medical Sciences.

## Conflict of Interests

The authors have no conflicts of interest to disclose.
